# Effects of midodrine and L‐NAME on systemic and cerebral hemodynamics during cognitive activation in spinal cord injury and intact controls

**DOI:** 10.14814/phy2.12683

**Published:** 2016-02-11

**Authors:** Jill M. Wecht, Joseph P. Weir, Miroslav Radulovic, William A. Bauman

**Affiliations:** ^1^ VA RR&D National Center for the Medical Consequences of Spinal Cord InjuryJames J. Peters VAMCBronxNew York; ^2^ The Medical ServiceJames J. Peters VAMCBronx New York; ^3^ Department of MedicineThe Icahn School of Medicine at Mount SinaiNew YorkNew York; ^4^ Department of Rehabilitation MedicineThe Icahn School of Medicine at Mount SinaiNew YorkNew York; ^5^ Department of Health, Sport and Exercise SciencesThe University of KansasLawrenceKansas

**Keywords:** Blood pressure, cerebral blood flow, cognition, L‐NAME, Midodrine, NOS inhibition, spinal cord injury, tetraplegia

## Abstract

We previously showed that increases in mean arterial pressure (MAP) following administration of midodrine hydrochloride (MH) and nitro‐L‐arginine methyl ester (L‐NAME) resulted in increased mean cerebral blood flow velocity (MFV) during head‐up tilt in hypotensive individuals with spinal cord injury (SCI) and question if this same association was evident during cognitive activation. Herein, we report MAP and MFV during two serial subtraction tasks (SSt) given before (predrug) and after (postdrug) administration of MH; (10 mg), L‐NAME (1 mg/kg) or no drug (ND) in 15 subjects with SCI compared to nine able‐bodied (AB) controls. Three‐way factorial analysis of variance (ANOVA) models were used to determine significant main and interaction effects for group (SCI, AB), visit (MH, L‐NAME, ND), and time (predrug, postdrug) for MAP and MFV during the two SSt. The three‐way interaction was significant for MAP (*F* = 4.262; *P* = 0.020); both MH (30 ± 26 mmHg; *P* < 0.05) and L‐NAME (27 ± 22 mmHg; *P* < 0.01) significantly increased MAP in the SCI group, but not in the AB group. There was a significant visit by time interaction for MFV suggesting an increase from predrug to postdrug following L‐NAME (6 ± 8 cm/sec; *P* < 0.05) and MH (4 ± 7 cm/sec; *P* < 0.05), regardless of study group, with little change following ND (3 ± 3 cm/sec). The relationship between change in MAP and MFV was significant in the SCI group following administration of MH (*r* ^2^ = 0.38; *P* < 0.05) and L‐NAME (*r* ^2^ = 0.32; *P* < 0.05). These antihypotensive agents, at the doses tested, raised MAP, which was associated with an increase MFV during cognitive activation in hypotensive subjects with SCI.

## Introduction

Decentralized autonomic cardiovascular control in persons with spinal cord injury (SCI) often results in chronic hypotension and orthostatic hypotension (OH), particularly in individuals with high cord lesions (i.e., above T6). While most hypotensive individuals with chronic SCI remain asymptomatic and do not complain of symptoms associated with cerebral hypoperfusion (i.e., dizziness, lightheadedness, blurry vision, nausea, etc.), evidence of reduced resting cerebral blood flow (CBF) has been reported in association with low systemic blood pressure (BP) in the SCI population (Yamamoto et al. 1980; Catz et al. 2007; Wilson et al. 2010); although controversy exists (Sahota et al. 2012; Phillips et al. 2014a). We recently found that middle cerebral artery CBF velocity (CBFv) was not increased during cognitive testing in individuals with SCI (Wecht et al. 2012), and noted significantly impaired memory and marginally impaired attention processing in hypotensive individuals with SCI compared to a normotensive SCI cohort (Jegede et al. 2010). Given the potential adverse impact of asymptomatic hypotension on CBF and cognitive function in the SCI population treatment options for low BP should be considered. However, we reported disparity between the evidence of hypotension (≈40%) and the clinical diagnosis (<1%) and treatment (<1%) in veterans with SCI (Wecht et al. 2013b; Zhu et al. 2013), which we attribute to the lack of evidence documenting the safe and effective use of antihypotensive agents to raise BP and improve CBF and cognitive performance in the SCI population.

In the United States, there are currently two FDA approved medications available for treatment of symptomatic neurogenic OH, an alpha‐agonist, midodrine hydrochloride (MH) and the norepinephrine precursor, L‐Threo‐3,4‐Dihydroxyphenylserine (droxidopa). We documented safe and effective increases in BP in hypotensive individuals with SCI following a single dose of MH (10 mg) (Wecht et al. 2010, 2011) and droxidopa (400 mg) (Wecht et al. 2013a). In addition, we reported that intravenous infusion of a nitric oxide synthase (NOS) inhibitor, nitro‐L‐arginine methyl ester (L‐NAME: 1.0 mg/kg) increased supine and orthostatic BP (Wecht et al. 2008, 2009); however, L‐NAME is an experimental antihypotensive agent and does not have FDA approval. Importantly, we noted a direct association between the orthostatic change in BP and CBFv following administration of MH and L‐NAME in hypotensive individuals with SCI (Wecht et al. 2010, 2011); however, we have not reported the effects of these antihypotensive agents on BP and CBFv during cognitive testing, and have not compared the BP and CBFv responses to MH and L‐NAME between individuals with SCI and able‐bodied (AB) controls.

The purpose of this investigation was to compare BP and CBFv responses between hypotensive individuals with SCI and AB controls (with low BP) during cognitive activation, using two serial subtraction tasks (SSt), one before (predrug) and one after (postdrug) administration of MH, L‐NAME, and no‐drug (ND). A secondary aim was to determine if changes in BP were correlated with changes in CBFv from predrug to postdrug following administration of the antihypotensive agents (MH and L‐NAME). We hypothesized that the BP and CBFv responses to MH and L‐NAME would be augmented in the SCI compared to the AB group and that changes in BP would relate to changes in CBFv in those with SCI.

## Materials and Methods

### Subjects

All subjects (*n* = 25) were between the ages of 22 and 61 years with no known history of CVD, pulmonary disease, or diabetes mellitus. Subjects were current nonsmokers for a minimum of 1 year prior to investigation and were not taking medications known to affect autonomic cardiovascular function. Subjects were instructed to be well hydrated and to avoid heavy exertion, caffeine, and alcoholic beverages for a minimum of 24 h prior to testing. The Institutional Review Board for Human Studies of the James J. Peters Veterans Affairs Medical Center granted approval, and informed consent was obtained prior to study.

### Study procedures

Subjects visited the laboratory on three separate occasions and performed an SSt before and after administration of L‐NAME (1.0 mg/kg), MH (10 mg), or ND. Study visits, although administered in random order, were not blinded because L‐NAME (Clinalfa, Hauptstrasse 144, 4416 Bubendorf, Switzerland) was administered intravenously via an anticubital catheter, MH (Mylan Pharmaceuticals Inc. 3711 Collins Ferry Road Morgantown, WV 26505) was administered orally with a glass of water and nothing was administered on the ND visit. Subjects arrived at the laboratory approximately the same time of day on all three study visits and remained seated for the duration of testing. Prior to data collection, a 20‐min period of quiet rest was provided in a thermoneutral, dimly lit room while the subjects were instrumented with ECG electrodes, BP monitors, and a head harness to secure probe placement for transcranial Doppler (TCD) ultrasound recording of the left middle cerebral artery (MCA). Immediately following the quiet rest period, and prior to drug administration, subjects were asked to perform the SSt (predrug) while simultaneous beat‐to‐beat heart rate (HR), BP, and CBF data were collected. At the completion of the predrug SSt the drug administration period was initiated – which varied based on the randomly selected visit order and included: (1) insertion of an intravenous catheter in the left antecubital vein for the 60‐min L‐NAME infusion (20–80 min); (2) oral administration of MH (40 min after the predrug SSt) or (3) quiet seated rest (ND visit). A second SSt (postdrug) was performed 90 min after completion of the predrug SSt, while simultaneous beat‐to‐beat HR, BP, and CBF data were collected.

### Heart rate & blood pressure monitoring

Instrumentation included continuous monitoring of the inter‐beat‐interval (IBI: msec) of HR using a standard electrocardiogram (Ivy Biomedical Systems Inc. 11 Business Park Drive Branford, CT 06405‐2959). The ECG signal was recorded from a 3‐lead configuration; lead site preparation was performed according to clinical standards and electrodes were placed at the distal right and left clavicle and in the left lateral 5th intercostal space (V‐5). Beat‐to‐beat finger BP (mmHg) was continuously monitored at the right middle finger using photoplethysmography (Finometer ^®^ MIDI Model‐2; Finopres Medical Systems B.V. Amsterdam, the Netherlands). To minimize interday and intraday variability in the finger BP assessments the photoplethysmographer was calibrated to brachial pressure immediately (≈30 sec) before each SSt and the arm was supported horizontal to the level of the heart on all three study visits. The systemic hemodynamic variables of interest included IBI, SBP, DBP, and mean arterial pressure (MAP: the primary outcome variable), which was calculated from finger BP as: (SBP + DBP + DBP)/3.

### Cerebral blood flow monitoring

Cerebral blood flow was estimated from blood flow velocity (FV) assessed at the left MCA using TCD ultrasound technology (Terumo Cardiovascular Systems 1311 Valencia Avenue Tustin, CA 92780‐6447). The left MCA was isonated because prior work suggests increased activation with respect to a baseline period during the SSt (Duschek and Schandry 2006). The TCD probe was operated at a frequency of 2.0 MHz to visualize the MCA and insonation was through the left temporal window. The MCA was identified by the target depth (45–55 mm), sound and direction of flow (i.e., toward the probe), the characteristic spectral waveform, relatively faster FV compared to other cerebral vessels, and by compression of the common carotid artery which resulted in an appropriate reduction in MCA flow velocity. Once the MCA was visualized, a head‐harness was used to secure probe placement for the duration of testing. Data collection output from the TCD monitor included systolic FV (SFV: cm/sec), diastolic FV (DFV: cm/sec) and mean FV (MFV: cm/sec); the primary outcome variable was MFV.

### Signal acquisition

The ECG, photoplethysmography, and transcranial Doppler signals were sampled at 500 Hz and were stored on a hard‐drive for subsequent analysis using customized data analysis programs written with LabVIEW graphical software for instrumentation (National Instruments, 11500 North Mopac Expressway Austin, TX 78759‐3504).

### Cognitive activation

The SSt is an easy to perform, brief assessment of information processing speed (Williams et al. 1996). Subjects were asked to subtract a single‐digit number from a three‐digit number in succession for 60 sec. Each SSt began at different three‐digit starting points and the number being subtracted varied randomly. For instance, subjects may have been asked to subtract the number 7 from 760 and on the next task may have been asked to subtract 6 from 801. The total number of attempts and the number of correct responses on the predrug SSt were compared between the SCI and AB groups and the change (from predrug to postdrug) in total attempts and number of correct responses was recorded following L‐NAME and MH administration. Hemodynamic data were collected continuously during the predrug and postdrug SSt and are reported as the average IBI, BP, and CBFv during the cognitive activation periods.

### Data analysis

Data are reported as mean ± standard deviation and were analyzed using a statistical analysis program (IBM SPSS Statistics 21). Subject characteristic data and performance on the predrug SSt were compared between the groups using unpaired t‐tests. Factorial analysis of variance (ANOVA) was used to determine main and interaction effects for group (SCI, AB) and visit (L‐NAME, MH, ND) for the predrug hemodynamic and cognitive data. To assess the effects of the drug intervention on the primary outcome variables of MAP and MFV separate univariate three‐way mixed factorial ANOVA models were constructed to determine significant interaction and main effects for group (SCI; AB), visit (L‐NAME; MH; ND), and time (predrug; postdrug). Follow‐up procedures were employed depending on the results of the factorial ANOVAs, and the Huynh–Feldt correction was applied where appropriate. Simple linear regression models were created for each group to determine the relationship between the change in MAP and change in MFV from predrug SSt to postdrug SSt for the MH and L‐NAME visits. Statistical significance was set at the 0.05 alpha level for all comparisons.

## Results

### Study subjects

Subjects with SCI (*n* = 15) were matched for demographic characteristics to the AB controls (*n* = 10); however, finometer BP recordings were unusable in one AB participant; therefore results are presented in nine control subjects. Subject demographic data are presented (Table 1); height was significantly lower in the AB compared to the SCI group. The study sample was largely male (75%), 11 were veterans (44%), and the sample was predominantly Caucasian (52%). Subjects with SCI were chronically injured (range: 1–42 years), mostly cervical lesions (80%), and two‐thirds were motor complete injuries (AIS A & B).

**Table 1:** Subject characteristics

	SCI	AB	*P* value
	*n* = 15	*n* = 9	
Age (years)	43 ± 12	36 ± 12	0.181
HT (cm)	176 ± 7	168 ± 9	0.040
WT (kg)	70 ± 10	65 ± 11	0.293
BMI (kg/m^2^)	22.8 ± 3.6	22.9 ± 2.8	0.911
Female Gender (*n*)	2	3	
Duration (years)	13 ± 12		
Level of SCI	C3‐T4		
Complete AIS A	5 (33%)		

HT, height; cm, centimeters; WT, weight; kg, kilograms; BMI, body mass index.

### Serial subtraction task

Although the total number of attempts on all (i.e., 3 per subject) of the predrug SSt did not differ between the AB and SCI groups (54.2 ± 27.4 vs. 44.5 ± 27.0, respectively; *P* = 0.154) the total number of correct responses on the predrug SSt was significantly higher in the AB compared to the SCI group (49.3 ± 28.8 vs. 38.3 ± 27.6, respectively; *P* = 0.047). However, there was no evidence of improved performance on the postdrug SSt in either group, regardless of study visit (data not shown).

### Hemodynamics predrug

There were no differences between study visits for the predrug hemodynamic data; therefore, the average predrug hemodynamic data for the three‐study visits are presented (Table 2). The group main effect was significant for SBP, MAP, DFV, and MFV, indicating that, regardless of study visit, BP and CBFv were reduced during the predrug SSt in the SCI compared to the AB group.

**Table 2:** Predrug hemodynamics

	SCI	AB	*P* value
IBI (msec)	790 ± 160	767 ± 126	0.717
SBP (mmHg)	92 ± 22	119 ± 30	0.009
MAP (mmHg)	68 ± 19	82 ± 15	0.021
DBP (mmHg)	54 ± 17	63 ± 13	0.075
SFV (cm/sec)	65 ± 14	80 ± 23	0.053
MFV (cm/sec)	37 ± 10	52 ± 15	0.005
DFV (cm/sec)	23 ± 10	33 ± 12	0.023

IBI, interbeat interval; SBP, systolic blood pressure; DBP, diastolic blood pressure; MAP, mean arterial pressure; SFV, systolic flow velocity; MFV, mean flow velocity; DFV, diastolic flow velocity.

### Change in systemic hemodynamics

**Figure 1:**
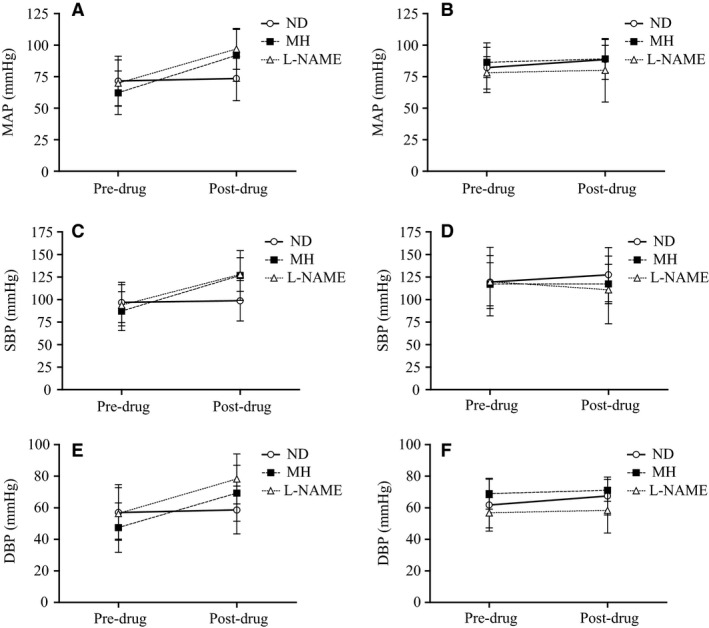
Change from predrug to postdrug following L‐NAME (open triangles), MH (closed squares) and ND (open circles) for MAP (top panels), SBP (middle panels), and DBP (bottom panels) in the SCI (A, C, E) and AB (B, D, F) groups.

Change in BP from predrug to postdrug is shown for the two study groups during the three study visits (Figure 1). The three‐way interaction effect was significant for MAP (*F* = 4.262; *P* = 0.020), such that MAP was increased in the SCI group (Figure 1A) from the predrug SSt to the postdrug SSt following MH and L‐NAME administration, but was flat following ND and regardless of study drug in the AB group (Figure 1B). Specifically in the SCI group, the change in MAP from predrug to postdrug was significant following MH (30 ± 26 mmHg, *P* = 0.011; 95% CI = 15 to 44 mmHg) and L‐NAME (27 ± 22 mmHg, *P* = 0.003; 95% CI =15 to 39 mmHg), and the magnitude of effect was comparable between the two antihypotensive agents (mean difference MH vs. L‐NAME = 3 mmHg; 95% CI = −21 to 16 mmHg). Compared to the ND condition in the SCI group, both drugs increased MAP from predrug to postdrug (MH vs. ND: mean difference 28 mmHg; 95% CI for difference = 7 to 48 mmHg and L‐NAME vs. ND: mean difference 25 mmHg, 95% CI for difference = 10 to 41 mmHg). Similar findings were evident for SBP and DBP (Figure 1C–F).

### Change in cerebral hemodynamics

**Figure 2:**
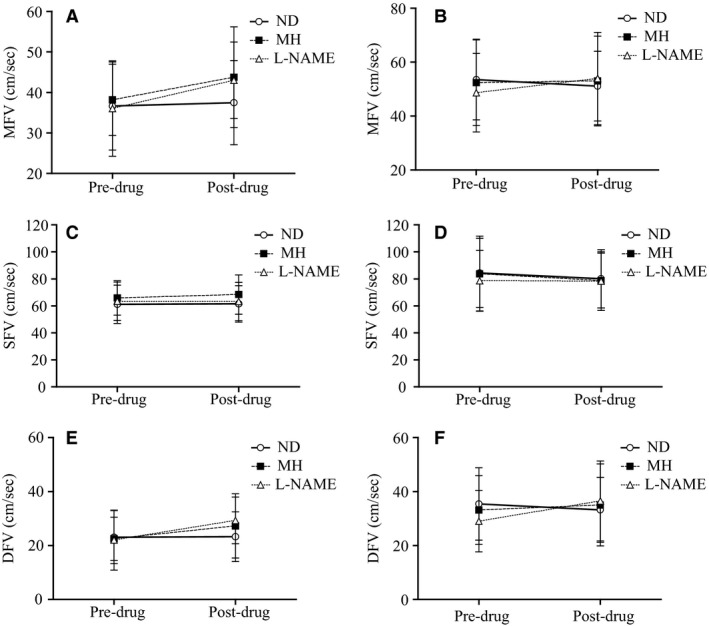
Change from predrug to postdrug following L‐NAME (open triangles), MH (closed squares) and ND (open circles) for MFV (top panels), SFV (middle panels), and DFV (bottom panels) in the SCI (A, C, E) and AB (B, D, F) groups.

The change from predrug to postdrug in CBFv is presented for the three study visits in the SCI and AB groups (Figure 2). Although the three‐way interaction effect was not significant for MFV (Figure 2A, B) the visit by time interaction was significant. The data indicate an increase in MFV in both groups from the predrug SSt to the postdrug SSt following L‐NAME (6 ± 8 cm/sec; 95% CI = 3 to 9 cm/sec) and MH (4 ± 7 cm/sec; 95% CI = 1 to 7 cm/sec), with little change following ND (3 ± 3 cm/sec; 95% CI = −3 to 3 cm/sec). Although follow‐up procedures indicated that the magnitude of change was not significantly different between L‐NAME and MH (95% CI for difference = −1 to 6 cm/sec), L‐NAME increased MFV from predrug to postdrug relative to the ND condition (L‐NAME vs. ND: mean difference = 6 cm/sec; 95% CI for difference = 2 to 11 cm/sec), whereas MH did not quite reach significance (MH vs. ND: mean difference = 4 cm/sec; 95% CI for difference = −0.2 to 8 cm/sec). Increase in MFV during the postdrug SSt was largely attributable to changes in DFV (Figure 2E, F), because, regardless of study visit there were no significant effects for SFV (Figures 2C, D).

### Relationship between MAP & MFV

**Figure 3:**
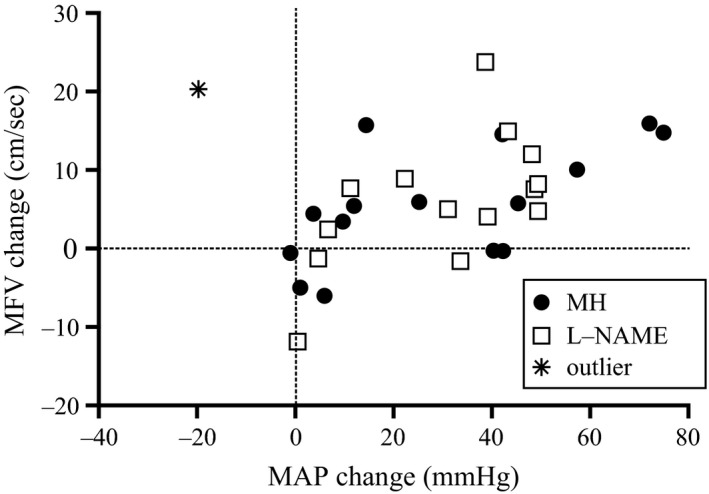
Relationship between change in MAP and change in MFV from predrug to postdrug during the SSt following MH (closed circles) and LN (open squares) in the SCI group. The slope of this relationship was significant for MH (*r* ^2^ = 0.39; *P *< 0.01) and LN (*r* ^2^ = 0.33; *P* < 0.05).

The relationship between change in MAP (mmHg) and change MFV (cm/sec) is depicted for the SCI group (Figure 3). Change in MFV was significantly associated with change in MAP following administration of L‐NAME (*r* = 0.57, slope = 0.26 cm × sec^−1 ^× mmHg^−1^; *P* = 0.03) and MH (*r* = 0.62; slope = 0.18 cm × sec^−1^ × mmHg^−1^; *P* = 0.01). For the L‐NAME analyses, one outlier was identified using both Mahalanobis distance (criterion = distance > the mean distance +3 times the standard deviation of the Mahalanobis distance) as well as having a Cook's distance = 3.72 (reflecting a data point with undue influence on the regression calculations). The outlying data point is indicated in Figure 3 (*) and the regression line was generated without this outlier, using an *n* = 14.

## Discussion

The primary aim of this investigation was to determine BP and CBFv responses to MH and L‐NAME in hypotensive individuals with SCI compared to AB controls during cognitive activation. The data suggest that these antihypotensive agents, at the doses tested, increased MAP in the SCI group but had no effect on MAP in the AB group. However, the effect of these antihypotensive agents on CBFv was mixed. Regardless of group affiliation MFV was increased following L‐NAME administration, whereas MFV was increased following MH administration in the SCI group alone. A secondary aim was to determine if there was an association between the change in BP and the change in CBFv from the predrug SSt to the postdrug SSt. As anticipated, change in MAP correlated with change in MFV following administration of either L‐NAME or MH in the SCI group.

### Predrug hemodynamics: cognitive function

Blood pressure and cerebral blood flow velocity were uniformly lower in the hypotensive individuals with SCI compared to AB controls during the predrug SSt and subjects with SCI responded with fewer correct answers than the AB controls. However, by study design, all subjects with SCI were hypotensive, and therefore we cannot determine if the low BP contributed to the poor performance on the predrug SST or if the SCI per se was contributory. That said, our previous findings suggest that hypotensive individuals with SCI perform more poorly than normotensive individuals with SCI on cognitive tests assessing memory (California Verbal Learning Test) and attention processing (Oral Trails A) (Jegede et al. 2010), and we have shown that individuals with SCI, regardless of the level of lesion (C4‐T10), do not appropriately adjust CBFv during cognitive testing (Stroop test), which related to poor test performance compared to AB controls (Wecht et al. 2012). The SSt assesses complex attention and speed of information processing (Williams et al. 1996), and although diabetics scored more poorly than nondiabetics, scores on the SSt did not differ between individuals with and without hypertension (Hawkins et al. 2011). Therefore, blood pressure may not contribute to performance on the SSt, which could have accounted for the lack of improvement in scores following BP elevation in our subjects with SCI. Future studies should investigate the effects of BP elevation on memory in hypotensive individuals with SCI.

### Hypertensive effect: SCI versus AB

These antihypotensive agents increased seated BP from the predrug SSt to the postdrug SSt in subjects with SCI, but had no effect in AB controls. Individuals with high level SCI (T4 and above), have some degree of decentralized peripheral sympathetic vasomotor control, which results in frankly low levels of resting plasma norepinephrine (NE).(Wecht et al. 2008; Wilson et al. 2010) It is believed that the prolific hypotension reported in individuals with high level SCI is a consequence of low plasma NE (Claydon and Krassioukov 2006), and that the responsiveness to antihypotensive therapy reflects inadequate baroreceptor buffering of BP (Wecht et al. 2008). In contrast, we believe that the healthy AB controls were able to buffer the rise in BP following administration of these antihypotensive agents by withdrawing tonic peripheral sympathetic vasomotor control (i.e., NE), as we previously demonstrated in response to L‐NAME infusion (Wecht et al. 2008). Moreover, the increase in BP was comparable following administration of L‐NAME (NOS inhibitor) and MH (alpha‐agonist) in the SCI group, suggesting that the BP response was independent of the mechanism responsible for the hypertensive response.

### Pressure flow relationship: autonomic impairment

The statistically significant relationship between change in MAP and change in MFV in the SCI group suggests that decentralized sympathetic cardiovascular control may alter the systemic pressure‐cerebral flow relationship. There is evidence of a passive relationship between systemic BP and CBF in several models of impaired peripheral sympathetic vascular control. Using positron emission tomography in individuals with autonomic failure secondary to Shy‐Drager syndrome a 19% increase in BP was associated with a 22% increase in CBFv (Ogawa et al. 1998) and superior cervical sympathetic nerve stimulation caused an increase in MCA flow velocity, which was directly related to an increase in systemic BP in three patients undergoing surgery for palmar hyperhidrosis.(Wahlgren et al. 1992) Moreover, pharmacologic manipulation of adrenergic sympathetic vascular control during rapid changes in BP secondary to the Valsalva maneuver (Zhang et al. 2004a) and thigh cuff deflation (Ogoh et al. 2008) demonstrate alteration in CBFv that differed significantly from a control condition. This evidence suggests that the autonomic nervous system may play a role in maintaining the relationship between systemic BP and CBFv and when autonomic regulatory control is altered, the pressure‐flow relationship may be impaired. While sympathetic adrenergic impairment may be a pathophysiological adaptation during rapid changes in BP, this altered pressure‐flow relationship may serve to improve CBFv following antihypotensive treatment in the SCI population.

### Pressure flow relationship: L‐NAME versus MH

There is conflicting evidence describing the effects of increase in BP on CBFv following administration of alpha‐agonists or NOS inhibitors. Increased MFV has been reported concurrent with BP elevation following administration of MH in models of essential hypotension, (Duschek et al. 2007a, b) in hemodialysis patients with orthostatic hypotension, (Fujisaki et al. 2007) and, following administration of L‐NAME a transient increase in CBF was reported in rats (Koskinen and Koch 2003). In addition, we previously reported a significant association between the change in MAP and MFV from supine to 45° head‐up tilt in hypotensive individuals with SCI (Wecht et al. 2011). In contrast, Phillips et al., reported significant increases in seated (Phillips et al. 2014b) and supine (Phillips et al. 2014a) MAP following administration of MH (10 mg); with no effect on MCA CBFv in ten individual with SCI (C4‐T5). In addition, Zhang et al. reported comparable increases in MAP following administration of the NOS inhibitor L‐NMMA (N^G^‐monomethyl‐L‐arginine) and an *α*_1_‐agonist (phenylephrine) in healthy AB controls, but neither agent increased CBFv (Zhang et al. 2004b).

While neither the alpha agonist nor the NOS inhibitor significantly raised MAP in the AB controls, L‐NAME appeared to increase MFV. This finding is in contrast to prior work demonstrating that although supine MAP was increased by 13% following systemic administration L‐NMMA CBFv remained unchanged (Zhang et al. 2004b). We speculate that the MFV response to L‐NAME reflects suppressed tonic inhibition of extra‐cranial sympathetic regulation of CBF by nitric oxide, independent of BP (Willie et al. 2014). An alternative explanation is that NOS inhibition may have reduced the diameter of the MCA (Stewart et al. 2013), which artificially increased TCD recording of flow velocity, but may not be indicative of a true increase in CBF.

### Clinical side‐note

It should be noted that the MAP and MFV responses to the study drugs varied among the individual participants with SCI. Six subjects with SCI responded with an equivalent rise in MAP following L‐NAME and MH, five subjects responded better to L‐NAME and four responded better to MH. While seven subjects with SCI had a similar increase in MFV following L‐NAME and MH administration, four subjects responded better to L‐NAME, and two subjects responded better to MH; two subjects did not respond with an increase in MFV following administration of either L‐NAME or MH. Furthermore, it must be appreciated that although the relationship between MAP and MFV in the SCI group was significant, change MAP accounted for only about 1/3rd of the variance in the change in MFV, which probably relates to the variability in individual response to these different antihypotensive agents. Taken together, these data suggest that, analogous to treatment of hypertension, individual responses to antihypotensive therapy vary and therefore development of a pharmacological armamentarium for the safe and effective treatment of hypotension in the SCI population is important.

### Limitations

Doppler ultrasound of the MCA assumes that vessel diameter remains constant throughout testing; however, we cannot account for the potential differential influence of these antihypotensive agents on the diameter of the MCA. In fact, there is recent evidence suggesting that administration of a nitric oxide donor (sodium nitroprusside) increases the diameter of the MCA (Stewart et al. 2013); therefore, systemic administration of L‐NAME, may have influenced MCA diameter thereby contributing to changes in CBF independent of changes in flow velocity or BP. We recently reported poor day‐to‐day reproducibility of finger BP assessments in the SCI and AB populations; however, we were unable to collect brachial BP during the 60 sec SSt, due to the short duration of the test and distraction to subjects; therefore, the BP data should be interpreted with caution. Conclusions regarding the ability to improve cognitive performance by increasing BP or CBFv are still speculative and cannot be confirmed with these data. Finally, the clinical utility of L‐NAME is questionable because NOS inhibition is not presently available as an FDA approved agent for treatment of hypotension.

## Conclusion

Both L‐NAME and MH, at the doses tested, relieved the hypotension in subjects with SCI and attenuated group differences in CBFv during cognitive activation. Moreover, the findings suggest that increase in MAP was associated with increase in MFV in subjects with SCI. We believe that optimal BP control should involve ascertaining the effects of antihypotensive medications on CBF rather than treating solely to “normalize” BP. Additional data are needed to determine the dose and length of time required to normalize CBF, and improve cognitive performance following BP elevation and for the development a clinical armamentarium for the safe and effective treatment of asymptomatic hypotension in the SCI population.

## Conflict of interest

The authors declared no conflict of interest.
